# The occurrence and management of fluid retention associated with TKI therapy in CML, with a focus on dasatinib

**DOI:** 10.1186/1756-8722-2-46

**Published:** 2009-11-12

**Authors:** David Masiello, Gerry Gorospe, Allen S Yang

**Affiliations:** 1 Jane Anne Nohl Division of Hematology and Center for the Study of Blood Diseases, University of Southern California Medical Center, 1441 Eastlake Ave Suite 7317, Los Angeles, CA 90033, USA

## Abstract

Tyrosine kinase inhibitors (TKIs) like dasatinib and nilotinib are indicated as second-line treatment for chronic myeloid leukemia resistant or intolerant to the current first-line TKI imatinib. These are agents are well tolerated, but potent and as such should be monitored for potentially serious side-effects like fluid retention and pleural effusions. Here we present key clinical trial data and safety considerations for all FDA approved TKIs in context for effective management of fluid retention and pleural effusions. Altering the dasatinib regimen from 70 mg twice daily to 100 mg daily reduces the risk of pleural effusion for patients taking dasatinib. Should pleural effusion develop, dasatinib should be interrupted until the condition resolves. Patients with a history of pleural effusion risk factors should be monitored closely while taking dasatinib. Patients receiving imatinib and nilotinib are not without risk of fluid retention. All patients should also be educated to recognize and report key symptoms of fluid retention or pleural effusion. Pleural effusions are generally managed by dose interruption/reduction and other supportive measures in patients with chronic myeloid leukemia receiving dasatinib therapy.

## Introduction

Chronic myeloid leukemia (CML) is a hematopoietic stem cell malignancy with an age-adjusted incidence rate of 1.5 per 100,000 individuals per year within the United States, accounting for 15% of all adult leukemias [1,2]. The median age of diagnosis is 66, but CML may occur in all age groups [1]. CML typically progresses through three sequential phases: chronic phase (CP), accelerated phase (AP), and terminal blast crisis (BC). Most often, patients are diagnosed during CP.

At the cellular level, CML is characterized by the presence of the Philadelphia (Ph) chromosome [3]. This genetic abnormality results from a reciprocal translocation between chromosomes 9 and 22, leading to the formation of the pathogenic tyrosine kinase signal transduction protein, BCR-ABL [4-6]. BCR-ABL is also found in some patients with acute lymphoblastic leukemia (Ph+ ALL).

If untreated, the prognosis for patients with CML is poor. Under these conditions the disease usually progresses from CP to BC within 3-5 years [2]. Even with the benefit of imatinib mesylate treatment, some patients with CML progress to BC [7]. Therefore, there is a strong medical need for effective treatments for this malignancy.

The treatment of CML was revolutionized by the use of tyrosine kinase inhibitors (TKIs) directed against BCR-ABL, the first developed being imatinib (Gleevec^®^). Currently, imatinib remains the only FDA-approved first-line treatment option for this disease [8]. Imatinib has been shown to benefit most patients; however, resistance and intolerance to this agent have emerged as clinical concerns. These problems may either prevent a patient from attaining a sufficient clinical response (suboptimal response), or may cause a patient to lose an existing one (relapse). In the pivotal phase III study of imatinib, 23% of patients faced initial, inherent (primary) resistance, and a further 4% of patients presented with intolerance to the agent [9,10]. After 7 years of follow-up, it was found that 40% of patients discontinued imatinib due to adverse events, lack of efficacy, bone marrow transplant, death, protocol violation, withdrawal of consent, loss of follow-up, or administrative reasons [11]. A large European retrospective survey found that 45% of all patients treated with imatinib displayed resistance or intolerance [12].

Reasons for imatinib resistance are multifactorial. The most understood mechanism is mutation of BCR-ABL, preventing imatinib from binding effectively to the protein [8]. It is thought to be the most important mechanism underlying secondary resistance. Other mechanisms include decreased intracellular levels of imatinib (caused by changed expression of drug efflux or influx proteins), increased levels of BCR-ABL (via gene amplification or over expression), or pathologic alteration of downstream intracellular pathways (e.g., SRC family kinases; SFKs).

Effective second-line treatments for imatinib-resistant or -intolerant patients with CML are now available. Dasatinib (Sprycel^®^) and nilotinib (Tasigna^®^) are both second-line TKIs approved for patients with CP or AP CML resistant or intolerant to imatinib. The drugs are similar in their ability to overcome resistance to imatinib therapy, but there are subtle differences in indications and side effect profiles that are worth mentioning. Nilotinib is associated with prolongation of the QT interval and therefore a screening EKG is recommended prior to starting therapy [13]. In addition, nilotinib administration requires the patient to fast prior to taking the twice daily dose. Dasatinib does not have a fasting or screening EKG requirement, but is associated with a higher incidence of pleural effusions [14]. Dasatinib is also indicated for the treatment of patients with BC CML or Ph+ ALL and who are resistant or intolerant to imatinib. It is important to note there are no direct comparisons of efficacy of nilotinib and dasatinib in CML.

Although both second-line TKIs are well tolerated, side effects do occur during treatment. Management of side effects is essential to ensure that patients continue treatment and have the best possible chance of a positive long-term outcome. In this review, we will focus on the the occurrence and appropriate management of pleural effusions during dasatinib therapy.

## Dasatinib

Dasatinib is a thiazole carboximide with potent activity against BCR-ABL and also SFKs [15]. This agent has 325-fold greater activity against unmutated BCR-ABL *in vitro *than imatinib, and displays activity in all but one of the known imatinib-resistant BCR-ABL mutations (i.e., T315I) [15-17]. Dasatinib has been demonstrated to be active and well tolerated in patients with imatinib resistance or intolerance across all phases of CML [18-20]. The current approved doses are 100 mg once daily for patients with CP CML, and 140 mg once daily for patients with advanced (AP or BC) CML or Ph+ ALL. Dasatinib is available in 20 mg, 50 mg, 75 mg, and 100 mg tablets, and may be swallowed whole, with or without a meal [13].

Dasatinib was originally approved across all phases of CML at a dosage of 70 mg twice daily. In key phase II studies, marked responses were attained across all phases of CML using this schedule. For example, after a minimum follow-up of two years, complete cytogenetic responses (i.e., Ph was undetectable) were reached in more than half (53%) of patients with CP CML [21]. These responses were mirrored by impressive rates of progression-free survival (80%) and overall survival (94%).

The recommended starting dose for patients with CP CML was changed from 70 mg twice daily to 100 mg once daily. This change was prompted by the results of a phase III dose optimization study in which the 100 mg once-daily dose demonstrated improved tolerability, plus insignificantly changed efficacy, compared with the previously recommended 70 mg twice-daily dose (discussed further below) [22]. The recommended starting dose for patients with advanced phase CML or Ph+ ALL remains 70 mg twice daily.

## The Toxicities of Dasatinib

The side effects associated with dasatinib therapy are predominantly mild or moderate (grade 1 or 2 by the National Cancer Institute Cancer Therapy Evaluation Program criteria), and are self-limiting or resolve following supportive care [18-20,22]. Dasatinib is associated with correspondingly positive rates of treatment compliance and toxicity-related withdrawal [18-20	,22].

The phase III dose-optimization of dasatinib study showed that the incidence of key treatment-related side effects can be reduced, while maintaining the efficacy of dasatinib, by manipulating the dosage schedule. After a minimum follow-up of 6 months, major cytogenetic responses were attained in 59% of patients receiving dasatinib 100 mg once daily and in 55% of patients receiving dasatinib 70 mg twice daily [22]. Concomitantly, incidences of severe (grade 3-4) side effects were significantly reduced in the 100 mg once-daily arm compared with the 70 mg twice-daily arm (30% vs. 48%; p = 0.001). The most frequently occurring side effects of dasatinib are hematologic, as would be expected for a leukemia therapy. Notably, the number of patients experiencing grade 3-4 thrombocytopenia were also significantly lower in the 100 mg once-daily arm (22% vs. 37%; p = 0.004). The number of patients discontinuing dasatinib as a result of toxicity in the 100 mg once-daily arm were correspondingly lower (4% vs. 11%).

The recurrence of side effects associated with imatinib intolerance is minimal, indicating that there is a lack of cross-intolerance in patients presenting with imatinib intolerance. After 8 months of follow-up in the pivotal phase II study in patients with CP CML, 7% of patients with imatinib-intolerant CP CML discontinued treatment with dasatinib due to drug-related toxicity [20]. After a minimum of 24 months of follow-up, discontinuation rates for dasatinib in patients intolerant to imatinib due to hepatotoxicity (0%), rash (1%), and cytopenias (6%) remained low [23].

## Pleural Effusion

The incidences of grade 3-4 nonhematologic side effects in response to dasatinib treatment are minimal [18-20]. However, one of the more problematic nonhematologic side effects that can occur on dasatinib treatment is pleural effusion.

The incidence of grade 3-4 pleural effusion in patients with CP CML from collated studies was 4% (n = 1150) [13]. This side effect is more common in patients with advanced disease. Incidence rates for grade 3-4 pleural effusion is 5% (n = 502) in AP CML, 10% in myeloid BP CML, and 6% (n = 280) in lymphoid BP CML or Ph+ ALL (n = 250). Patients over the age of 65 years are more likely to experience fluid retention events, and should also be monitored closely [13]. The phase III dose-optimization study in patients with imatinib-resistant or -intolerant CP CML demonstrated that changing the dosage from 70 mg twice daily to 100 mg daily more than halves the incidence of any grade pleural effusion (16% vs. 7%, p = 0.024) (Table 1) [22].

**Table 1:** Incidence of pleural effusion in patients with CP CML with exposure to dasatinib

	Dosage		
		
	70 mg twice daily n = 167	100 mg once daily n = 166	P-value
Pleural effusion^a^			
All grades	26 (16%)	12 (7%)	0.024
Grades 3-4	2 (1%)	2 (1%)	-

^a^Classifications are as follows: grade 1, asymptomatic; grade 2, symptomatic, intervention with diuretics or up to two therapeutic thoracenteses indicated; grade 3, symptomatic and supplemental oxygen is required, greater than two therapeutic thoracenteses, tube drainage, or pleurodesis indicated; grade 4, life-threatening (e.g., causing hemodynamic instability); grade 5, death.

Data from Shah et al[22]

The mechanism underlying the development of pleural effusions during dasatinib therapy is currently unclear, and it is possible that pleural effusions are multifactorial [24]. Pleural effusions may be related to fluid retention resulting from nonspecific inhibition of platelet-derived growth factor receptor-β or other kinases [25]. There is also evidence that pleural effusions may be immune-related, as shown by lymphocytic infiltration of pleural fluids and an association between effusions and immune-mediated reactions, such as rash and autoimmune events [24,26]. It has been suggested that dasatinib may inhibit the function of normal T cells [27], and bind major regulators of the immune system [28]. Factors significantly related to the development of pleural effusion include a history of cardiac disease, hypertension, hypercholesterolemia, history of autoimmune disease, and history of skin rash during imatinib or dasatinib therapy [24,25].

Pleural effusions are potentially serious and must be treated promptly. To facilitate more rapid identification of pleural effusions, patients should also be educated to recognize and report relevant symptoms - i.e., chest pain, dyspnea and dry cough. In a study of patients who developed dasatinib-related pleural effusion (n = 48), all patients reported dyspnea at the time pleural effusion was reported [25]. The grade of dyspnea correlated with the radiographic extent of pleural effusion. Also, 29% of these patients also experienced pericardial effusion. Patients with a history of risk factors should be monitored closely, and measures, including optimizing blood pressure and serum cholesterol levels through medication, and performing a baseline chest x-ray, are also recommended.

**Figure 1:**
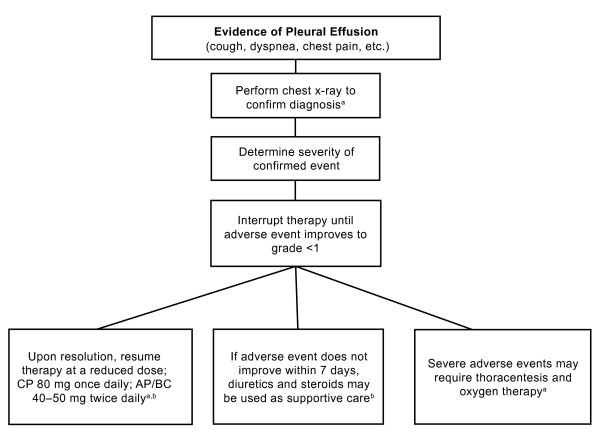
**The management of pleural effusion emerging on treatment with dasatinib**. ^a^BMS, 2009 [13]. ^b^NCCN, 2009 [2]. Abbreviation: ANC = absolute neutrophil count

Pleural effusions are generally managed by dose interruption/reduction, and supportive measures [2,25]. See Figure 1 for recommended management steps. Patients with CML exhibiting symptoms of pleural effusion should undergo radiographic testing. For confirmed incidences of pleural effusion, therapy should be interrupted until the event improves and later resumed at a reduced dose. The use of diuretics and steroids may be warranted. If an immune-related mechanism is indeed responsible for pleural effusion occurring during dasatinib treatment, corticosteroids are likely to be more effective than diuretics as an adjunct to dose reduction/interruption [26,29].

## Nilotinib

Nilotinib, a derivative of imatinib, was approved by the FDA in late 2007 for the treatment of adult patients with CP or AP CML resistant or intolerant to prior therapy with imatinib. The activity of nilotinib (400 mg orally twice daily) in patients with all phases of CML resistant or intolerant to imatinib has been confirmed in phase II studies [30-32]. In a trial of 280 patients with CP CML, a MCyR rate of 48% was observed after 6 months of follow-up [31]. At 12 months, the estimated survival was 95%. Cross-intolerance between nilotinib and imatinib was minimal in these studies.

While imatinib has been commonly associated with grade 1-2 fluid retention (59.9% in the multicenter phase III study that led to its approval), nilotinib is not as frequently associated with these events. Any-grade peripheral edema was reported in 11% of patients with CP CML receiving nilotinib, but none of these cases were severe [14]. Patients with AP CML experienced similar rates of edema. Pleural effusions is uncommonly associated with nilotinib therapy (1%) [30]. Management of these AEs is best treated with dose interruptions, and therapy can be resumed at the 400 mg once daily dose after resolution [14]. Besides fluid retention, other adverse events contribute to the safety profile of both imatinib and nilotinib, and HCPs should be familiar with these before initiating therapy.

## Conclusion

Currently three TKI therapies are available to patients with CML. Imatinib remains the recommended frontline therapy for patients with CP CML, but two new therapies, dasatinib and nilotinib, are available for CML patients who are resistant to or intolerant of imatinib therapy. Although the newer TKIs are similar, there are differences in their side-effects and indications. To date, there is no data comparing the efficacy of these three drugs directly, but these studies are currently ongoing. This article has focused specifically on the management of pleural effusions associated with dasatinib therapy. Fluid retention AEs have been associated with all three BCR-ABL inhibitors currently on the market, but pleural effusions may be more common with dasatinib therapy. These events are manageable, generally mild-to-moderate in severity, and occur more frequently in older patients (= 65 years) and/or patients with advanced CML disease.

The current recommended regimen of dasatinib for patients with CP CML is 100 mg once daily. This dose is associated with significantly fewer occurrences of key treatment-related side effects (including grade 3-4 pleural effusion) in comparison with the previously recommended regimen of 70 mg twice-daily dasatinib. Dasatinib 70 mg twice daily remains a highly effective treatment for patients with advanced CML and Ph+ ALL.

Clinical experience has shown that pleural effusions are generally reversible following a combination of dose interruption/reduction and additional supportive measures. In some rare cases more invasive steps like thoracocentesis or chest tubes are necessary to resolve the condition. In order to ensure appropriate management, patients should be vigilantly monitored for pleural effusions. Additionally, patients should be educated to recognize relevant symptoms of pleural effusions and other drug-related side effects and encouraged to report such symptoms to their physicians.

## Competing interests

Consultant or Advisory Role: DM, Novartis, and Bristol-Myers Squibb. GG, Bristol-Myers Squibb. ASY, Celgene, Eisai, and Vion. Honoraria: DM, Novartis, and Bristol-Myers Squibb. GG, Bristol-Myers Squibb. ASY, Bristol-Myers Squibb, Celgene, and Eisai. Stock ownership: ASY, TherEpi. Research Funding: GG, Bristol-Myers Squibb. ASY, Celgene, Novartis, and Methylgene.

## Authors' contributions

GG, DM, and AY contributed equally to the content and focus of the manuscript from its earliest conception. All authors read and approved the final edition.
